# High efficiency perovskite quantum dot solar cells with charge separating heterostructure

**DOI:** 10.1038/s41467-019-10856-z

**Published:** 2019-06-28

**Authors:** Qian Zhao, Abhijit Hazarika, Xihan Chen, Steve P. Harvey, Bryon W. Larson, Glenn R. Teeter, Jun Liu, Tao Song, Chuanxiao Xiao, Liam Shaw, Minghui Zhang, Guoran Li, Matthew C. Beard, Joseph M. Luther

**Affiliations:** 1 College of Chemistry, Nankai University, 300071 Tianjin, China; 2 National Renewable Energy Laboratory, Golden, CO 80401 USA; 3 Institute of New Energy Chemistry Material, Nankai University, 300350 Tianjin, China; 4 Warren Wilson College, Asheville, NC 28815 USA

**Keywords:** Solar cells, Quantum dots

## Abstract

Metal halide perovskite semiconductors possess outstanding characteristics for optoelectronic applications including but not limited to photovoltaics. Low-dimensional and nanostructured motifs impart added functionality which can be exploited further. Moreover, wider cation composition tunability and tunable surface ligand properties of colloidal quantum dot (QD) perovskites now enable unprecedented device architectures which differ from thin-film perovskites fabricated from solvated molecular precursors. Here, using layer-by-layer deposition of perovskite QDs, we demonstrate solar cells with abrupt compositional changes throughout the perovskite film. We utilize this ability to abruptly control composition to create an internal heterojunction that facilitates charge separation at the internal interface leading to improved photocarrier harvesting. We show how the photovoltaic performance depends upon the heterojunction position, as well as the composition of each component, and we describe an architecture that greatly improves the performance of perovskite QD photovoltaics.

## Abstract (web summary)

Metal halide perovskites have wide tunability in both material and device structure. Here Zhao et al. fabricate heterojunctions of colloidal perovskite quantum dots with different composition using layer-by-layer deposition and demonstrate improved photovoltaic performance with enhanced photocarrier harvesting.

## Introduction

Heterostructures within optoelectronic devices offer unique control of the electron and hole energy levels throughout the device^1–3^. For example, in photovoltaic (PV) devices, energy band engineering can enable better harvesting of photogenerated charge carriers^4–6^. This concept is especially prevalent in vapor deposited heterostructures using MOCVD and MBE of III–V semiconductors^7^  as well as  in sputtered or thermally evaporated systems such as CIGS where the composition at surfaces can be altered to promote a buried location for the actual *p-n* charge separating junction^8,9^. In solution processed metal halide perovskite semiconductors, such a concept has not been successfully demonstrated primarily due to there being no existing pathway to prevent the solvation of the first deposited perovskite layer by the solvent of the subsequently deposited perovskite layer. In contrast, for perovskite QD films, the solvent does not technically dissolve the precursor materials, but rather suspends the QDs by their surface ligands in a colloidal solution. In layer-by-layer deposition of perovskite QD films, a layer of QDs is deposited from the colloidal solution and then ligands are chemically removed, rendering the resulting film insoluble to the nonpolar solvent, thus, enabling the ability to process the subsequent QD-layers without affecting the underlying film^10–12^. In our standard process, a QD-colloid in hexane or octane contains perovskite QDs terminated with organic ligands and is first deposited onto the desired substrate. The resulting QD film is treated with methyl acetate (MeOAc) that effectively removes native oleate ligands^12^. A second QD deposition can then be applied and the ligand removal process is repeated. In principle, the second (or any subsequent) solution can contain either the same QDs or QDs of different composition allowing for the composition of the perovskite film to be modulated in almost any desired order throughout the film thickness.

## Results

### Demonstration of heterojunction

**Fig. 1:**
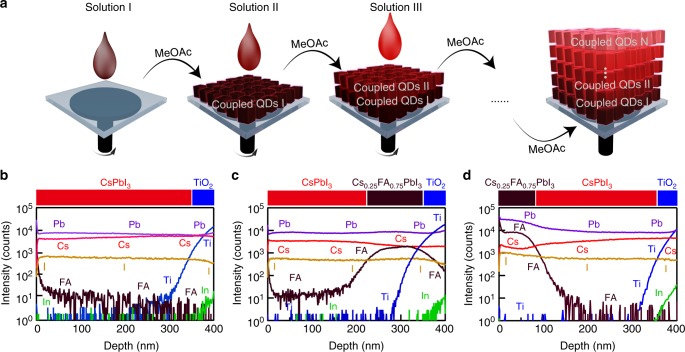
Heterojunction structure of QD films. **a** Schematic overview of layer-by-layer assembly showing a perovskite QD film composed of different layers of QDs. MeOAc treatment is carried out between the deposition of different QD layers to remove the native oleate ligands and to render the deposited QDs insoluble in the solvent. **b**–**d** ToF-SIMS depth profile of samples with the structure and interface location indicated in the bar above the plots of CsPbI_3_/TiO_2_ (**b**), CsPbI_3_/Cs_0.25_FA_0.75_PbI_3_/TiO_2_ (**c**), and Cs_0.25_FA_0.75_PbI_3_/CsPbI_3_/TiO_2_ (**d**). All relevant elements from perovskite QD layer and TiO_2_ are shown. Compared to the structure without Cs_0.25_FA_0.75_PbI_3_, the FA signal shows a sharp and significant change and the Cs signal also shows a slight change at the boundary between the CsPbI_3_ and Cs_0.25_FA_0.75_PbI_3_ QD layers. The In signal is shown as a reference for when the ToF-SIMS profiling beam reaches the ITO on the substrate

To demonstrate the compositional heterojunction (schematically shown in Fig. 1a) as described above, we employ time-of-flight secondary ion mass spectrometry (ToF-SIMS) depth profiling^13^ on various films prepared herein (Fig. 1b–d). The SIMS detects signals for Cs, formamidinium (FA), Pb, I, Ti, and In while sputtering through the film using an ion beam^13^. First, a pure CsPbI_3_ QD film on TiO_2_ is characterized and the Cs, Pb and I signals of the QD film are observed in the ToF-SIMS depth profile. (Fig. 1b) At roughly 300 nm into the film, a sharp rise of the Ti signal becomes prominent indicating the ion beam has reached the underlaying TiO_2_ layer. In Fig. 1c, we show ToF-SIMS for a heterostructured film containing CsPbI_3_ QDs deposited on top of Cs_0.25_FA_0.75_PbI_3_QDs. At roughly 200 nm into the film, the Cs signal decreases slightly while the FA signal rises (Fig. 1c). The distinct and sharp interface (rising several orders of magnitude) seen here with ToF-SIMS is nearly as sharp as the CsPbI_3_ interface with TiO_2_ in such a sample. In Fig. 1d, the perovskite compositions of the film structure are reversed compared to Fig. 1c, and here it is clear that a similar interface is formed within the film stack with the diminishment of the FA signal along with a slight increase in the Cs signal. This indicates a clear interface between layers of QDs with different compositions is formed and the sequence of these two QD layers can also be controlled via layer-by-layer deposition. In Supplementary Fig. 1 we show using ToF-SIMS that the integrity of such heterojunctions is minimally compromised after aging or moderate heating.

### Optical and electronic properties

**Fig. 2:**
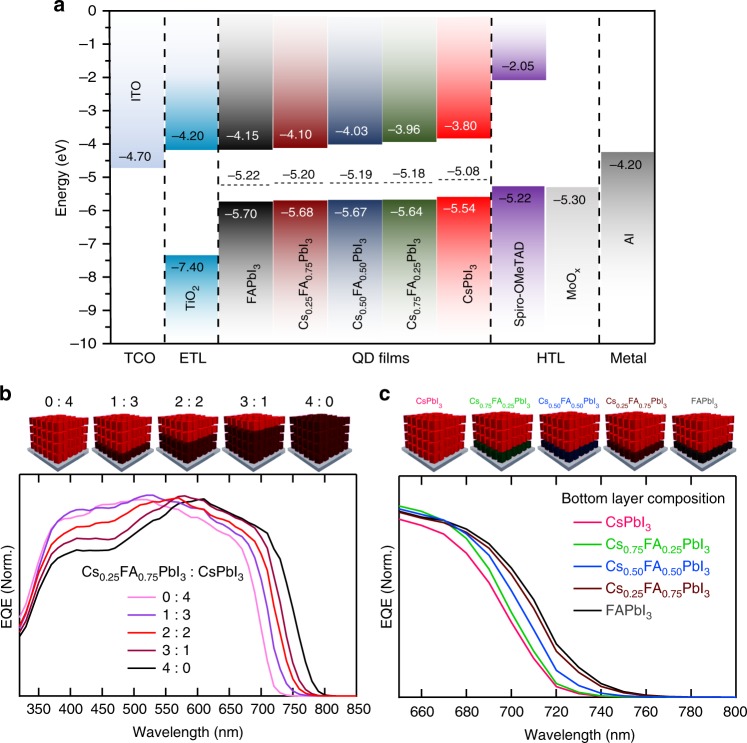
Optical and electronic properties of perovskite QD heterojunction. **a** Energy band positions for perovskite QD compositions and contact layers considered in this work. The Fermi level positions (denoted by horizontal dotted lines) and valence-band edges (*E*_v_) of all perovskite QD films were determined by UPS and XPS, respectively. The conduction band edges (*E*_c_) were calculated by adding the bandgap energy^26^ to *E*_v_ for the corresponding perovskite QD films. **b** EQE spectra of solar cells with varying thickness ratio of the Cs_0.25_FA_0.75_PbI_3_ layer to CsPbI_3_ layer in the perovskite QD absorber. **c** EQE spectra of solar cells with different compositions of the bottom layer in the perovskite QD absorber where the thickness ratio of the mixed-cation Cs_x_FA_1–x_PbI_3_ QD layers to CsPbI_3_ QD layers is 1:3 in all cases

The successful construction of perovskite heterostructured films allows the investigation of the resulting composite optoelectronic properties. First, the electronic properties of the heterostructure are studied with X-ray photoelectron and ultraviolet photoelectron spectroscopies (XPS and UPS) to probe energy band positions in films of each QD composition (see Methods and Supplementary Fig. 2 for details and analysis). In Fig. 2a, we plot the resulting energy level positions for each QD film. The measured energy levels indicate that a higher FA content leads to deeper conduction band edges, valence-band edges, and Fermi level positions relative to vacuum. Thus, there is an opportunity to create an energy offset that could drive electrons towards a layer containing more FA while holes are driven toward layers with less FA. The band positions for spiro-OMeTAD, TiO_2_ and contacts are also shown in Fig. 2a for reference.

Next, we determine how the macroscopic optical properties of the films are controlled by the varying thickness and composition of each layer. It has been previously shown that perovskite QD devices with ~300-nm-thick perovskite QD films have optimized power conversion efficiencies (PCEs)^10,14,15^. To be consistent, we perform a 4-cycle spin-coating procedure which, due to the QD concentration and solution properties, leads to a perovskite QD film with a total thickness of ~300 nm. Figure 2b presents the external quantum efficiency (EQE) of PV devices as a function of the internal heterostructure interface position of the Cs_0.25_FA_0.75_PbI_3_ portion to that of CsPbI_3_. Two main features to note are the systematic shift in the absorption onset from 720 nm for pure CsPbI_3_ films to 790 nm for a film containing only Cs_0.25_FA_0.75_PbI_3_ and a reduction in the collection efficiency of blue wavelengths for the devices containing higher amounts of FA within the film. In addition, the effect of changing the composition of lower-bandgap bottom layer in EQE is also investigated and presented in Fig. 2c. The composition of the bottom layer again causes a systematic shift in the absorption onset of the composite film but is less dramatic than observed in Fig. 2b.

### Device design and performance

**Fig. 3:**
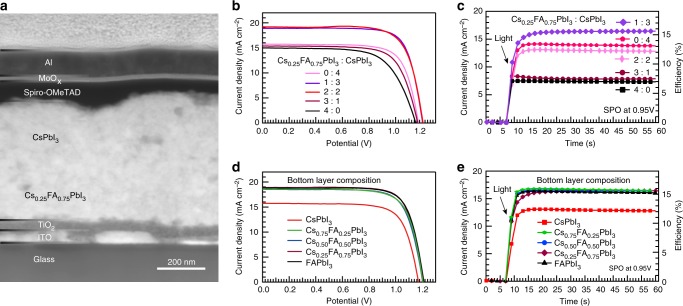
Photovoltaic performance of solar cells. **a** Cross-sectional STEM-HAADF image of devices with the structure of Glass/ITO/TiO_2_/Cs_0.25_FA_0.75_PbI_3_/CsPbI_3_/spiro-OMeTAD/MoO_x_/Al. **b**, **c** *J–V* curves recorded by reverse scans (from open circuit to short circuit) and SPO at 0.95 V of devices with different thickness ratio of Cs_0.25_FA_0.75_PbI_3_ layer to CsPbI_3_ layer in the perovskite QD absorber, respectively. **d**, **e** *J–V* curves measured via reverse scans and SPO at 0.95 V of devices with different compositions of the bottom layer in the perovskite QD absorber where the heterojunction position is constant

Given the optical properties of the heterojunction-containing films and the energetics shown above, Fig. 3 presents PV performance of the corresponding devices. Using the device architecture shown in Fig. 3a, a series of solar cells were fabricated (EDS elemental mapping of the cross section and morphology of QDs presented in Supplementary Figs. 3 and 4, respectively). Figure 3b shows *J–V* curves for devices to investigate the impact of thickness ratio of Cs_0.25_FA_0.75_PbI_3_ layer to CsPbI_3_ layer in the perovskite QD absorber. However, since we observe non-negligible hysteresis during the reverse and forward scans of devices (shown in Supplementary Fig. 5), the power conversion efficiency (PCE*)* from stabilized power output (SPO) at 0.950 V is reported (Fig. 3c). This figure of merit (PCE from SPO) has now become required for accurate reporting of efficiency for perovskite solar cells^16,17^. The reproducibility of these devices is demonstrated by showing the histograms of PCE from SPO (Supplementary Fig. 6). Despite similar PCE from the reverse scans for several devices, the device containing layer ratio of 1:3 (Cs_0.25_FA_0.75_PbI_3_: CsPbI_3_) has the highest PCE of 15.52% (see Table 1 and parameters from the forward scans presented in Supplementary Table 1 as well as heterojunction layer and device stability in the Supplementary Figs. 1 and 7). The improvement is mainly attributed to increased *J*_sc_ and suggests more photogenerated charge carriers are harvested with this architecture^18^. As a control experiment, devices from simply blending the two QD solutions at the same ratio (1:3 Cs_0.25_FA_0.75_PbI_3_: CsPbI_3_) were also prepared (Supplementary Fig. 8). It is anticipated that this would form more of a bulk heterojunction architecture rather than the planar heterojunction films described here. These bulk heterojunction QD devices show characteristics similar to single component devices rather than the improved carrier harvesting seen in planar heterojunction devices.

**Table 1:** Photovoltaic parameters of solar cells

Thickness ratio (Cs_0.25_FA_0.75_PbI_3_: CsPbI_3_)	*V*_oc_ (V)	*J*_sc_ (mA cm^−2^)	FF (%)	PCE (%)	SPO (%)
0:4	1.17	15.75	74	13.67	12.15
1:3	1.20	18.91	76	17.39	15.52
2:2	1.21	19.21	74	17.16	13.11
3:1	1.17	15.37	72	12.84	7.45
4:0	1.15	14.99	66	11.43	6.94

*J*–*V* parameters from reverse scans and conversion efficiency of devices with different thickness ratio of Cs_0.25_FA_0.75_PbI_3_ layer to CsPbI_3_ layer in the perovskite QD absorber

### Time-resolved carrier dynamics  of heterojunction films

To rationalize the high PCE of perovskite/perovskite QD heterojunctions, the mobility and lifetime of photogenerated carriers in each QD film were obtained using time-resolved microwave conductivity (TRMC). We find the carriers in the more FA-containing films possess longer free-carrier lifetimes but also exhibit lower effective mobility compared to pure CsPbI_3_ QD films (Supplementary Fig. 9). In other words, the trade-off in lower carrier mobility for longer average carrier lifetime results in increased recombination and less charge collection for thick FA-containing layers. Consequently, we observe a decrease in *FF* and *J*_sc_ for the devices with excessively thick FA-containing layers. This also helps to explain the reduction of EQE shown for 350 to 550 nm wavelengths in Fig. 2b. Light with wavelength around 450 nm is primarily absorbed in the FA-containing heterojunction component and shows poorer collection in thicker FA-containing layers. In Fig. 3d, e, we present *J–V* curves and SPO for devices with different compositions of the lower bandgap layer in the heterojunction with the optimized heterojunction position determined from Fig. 3c. There is negligible difference for device performance when the bottom-most layer of the absorber is Cs_0.75_FA_0.25_PbI_3_, Cs_0.5_FA_0.5_PbI_3_, Cs_0.25_FA_0.75_PbI_3_, or FAPbI_3_ (see Supplementary Table 2), which may be associated with the slightly different energy levels and that the fast charge separation is similar in each case.

**Fig. 4:**
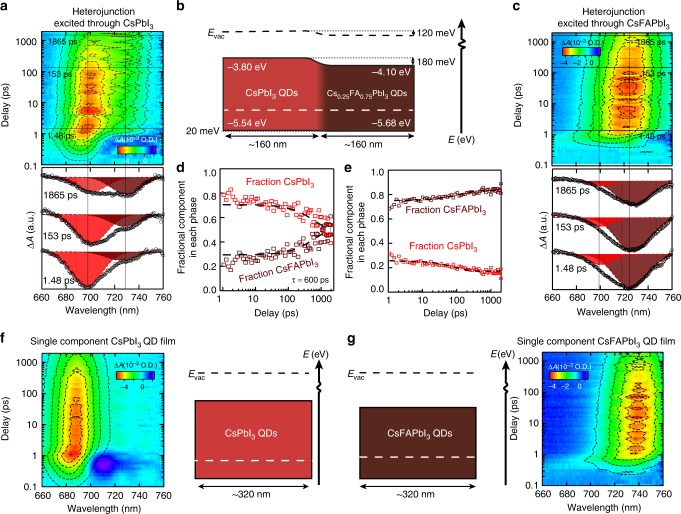
Transient absorption of heterojunction QD films. **a** Pseudocolor image of transient response for QD heterostructure when photoexciting through the CsPbI_3_ layer. For each of the surface plots with dashed contour lines one can visualize the carrier populations at various delay time delays between the pump and probe beam with the model described. Select cuts through the data are presented below the surface plots and offset vertically for clarity. Cuts are shown at 1.48 ps, 153 ps, and 1865 ps time delay. The spectra at each delay can be represented by the bleaching of two Gaussian peaks, one has a center wavelength at 697 nm (red-shaded Gaussian) and is associated with carriers occupying CsPbI_3_ while the other peak has a center wavelength at 728 nm (brown-shaded Gaussian) and is associated with carriers spatially occupying the Cs_0.25_Fa_0.75_PbI_3_ component. Vertical lines show the position of the two centered components. **b** Energy level diagram of the heterostructured film with estimated band alignment from UPS. In experiment **a** light impinges from the left (CsPbI_3_ QDs) while for **c** light impinges from the right (Cs_0.25_FA_0.75_PbI_3_ QDs). From the alignment, electrons are driven to the FA-containing side with a conduction band offset of 180 meV while holes have a small (20 meV) offset driving holes toward CsPbI_3_ **c** Pseudocolor image of transient response for QD heterostructure when photoexciting through the Cs_0.25_FA_0.75_PbI_3_ layer. The coloring scheme of the decomposed spectra is the same as in **a**. **d**, **e** The decomposed fraction of the TA signal for each delay measured that arises from the CsPbI_3_ (red-squares) and Cs_0.25_FA_0.75_PbI_3_ (brown-squares) as a function of time when photoexciting through CsPbI_3_ (**d**) or through Cs_0.25_FA_0.75_PbI_3_ (**e**). For **d**, the fraction of CsPbI_3_ decreases over time while Cs_0.25_FA_0.75_PbI_3_ increases as electrons are transferred into the FA-containing layer with a charge transfer time average of around 600 ps. For **e**, the fraction of CsPbI_3_ and Cs_0.25_FA_0.75_PbI_3_ is analyzed after the first ps and then stay relatively unchanged. **f** Pseudocolor image (left) and diagram (right) of a thick single component CsPbI_3_ film **g** Pseudocolor image (right) and diagram (left) of pure Cs_0.25_FA_0.75_PbI_3_

To better understand the charge separation dynamics induced by the designed heterojunction, we performed transient absorption (TA) spectroscopy which can independently probe carrier populations in each component of the heterojunction upon illumination. In the TA spectra (presented as pseudocolor images, Fig. 4) near the bandgap energy, a reduced absorption (ground state bleach) generally corresponds to the presence of excitons or free-charge carriers and the center of the reduced absorption indicates the exciton transition energy^19^. In Fig. 4 we show TA data for a heterojunction film excited at 400 nm either through the high bandgap CsPbI_3_ side (Fig. 4a) or through the lower bandgap Cs_0.25_FA_0.75_PbI_3_ side (Fig. 4c) as well as single component films of each composition (Fig. 4f, g). The anticipated energy band diagram is shown in Fig. 4b, indicating a 180-meV conduction band offset that will drive electrons towards the FA-containing component. The valence-band offset favors hole transfer to CsPbI_3_ but has only a small energetic offset of 20 meV, which is less than *kT* at room temperature, thus holes will mainly diffuse in the heterostructure. The TA spectra cannot be represented by only one bleaching component and we model our data by a sum of two Gaussian peaks, one centered at 697 nm and corresponds to carriers residing in the CsPbI_3_ component and another centered at 728 nm corresponding to carriers residing in the Cs_0.25_FA_0.75_PbI_3_ component. In Fig. 4a, a strong bleach is mainly observed at 697 nm due to excitation into the CsPbI_3_ component. As the delay between the pump and probe beam is swept between 1 ps and 2000 ps, the bleach component at 697 nm is transferred to one at 728 nm due to electron transfer from CsPbI_3_ to Cs_0.25_FA_0.75_PbI_3_. In Fig. 4d, the fractional component of the bleach in each component is plotted versus delay time and we find that initially 80% of the bleach is composed of that attributed to the CsPbI_3_ while ~20% initially penetrates through the CsPbI_3_ to Cs_0.25_FA_0.75_PbI_3_. The fractional component in the CsPbI_3_ decreases with time with a characteristic electron transfer time of ~600 ps. Such induced charge separation is advantageous for suppressing recombination and enhancing charge extraction^20,21^.

In Fig. 4e, the fractional components are plotted when the film is instead excited through the Cs_0.25_FA_0.75_PbI_3_ side. Again, about 20% of the excitation is transmitted through the Cs_0.25_FA_0.75_PbI_3_ to the CsPbI_3_ back layer in this case and the bleach signal remains roughly constant with time. In the lower panel in Fig. 4a, c, selected line cuts from the surface plots are shown along with component Gaussian fits to better visualize the charge transfer. The selected cuts in Fig. 4a show the increasing component of the bleach growing in at 153 ps and 1865 ps relative to 1.48 ps after excitation. In Fig. 4c, initially at 1.48 ps, a small 20% component of the bleach is due to the CsPbI_3_ layer as discussed but this signal decreases with increased pump-probe delay since TA has been shown to be more sensitive to electron than hole signatures^22^ and a much lesser driving force for hole transfer as indicated in the band diagram.

We also performed TA for pure single component CsPbI_3_ QD and pure Cs_0.25_FA_0.75_PbI_3_ (shown in Fig. 4f, g, respectively). These single component films show a bleach at 688 nm for CsPbI_3_ QDs and 738 nm for the Cs_0.25_FA_0.75_PbI_3_ sample which do not evolve much with time. These spectra are provided for reference. As explained by this TA analysis, charge transfer inside the absorber induced by heterojunction efficiently separates photogenerated electrons and (to a lesser degree) holes driving them opposite directions.

## Discussion

We develop perovskite devices that enables the rational design of charge separating interfaces within a perovskite QD absorber layer. By controlling the QD composition during layer-by-layer QD-film deposition, we can locate a spatial heterostructure arbitrarily within the resulting perovskite layer. We show how the composition of each heterostructure-component can be optimized based on charge-carrier lifetimes and mobilities within each component-QD to achieve a PCE from SPO up to 15.74%. Our concept could be easily employed with QDs and other forms of absorber layers (thin films^15^, 2D structures^23^, or combinations therein^24^). Furthermore, our approach should lead to a larger design-space which will impact a wider range of perovskite-based optoelectronic applications such as LEDs, transistor, (spintronic or quantum information processing, etc.).

## Methods

### Materials

Formamidinium iodide (CH(NH_2_)_2_, FAI) was purchased from Dyesol. Lead (II) iodide (PbI_2_; 99.9985%) and bis(trifluoromethane)sulfonimide lithium salt (Li-TFSI) were purchased from Alfa Aesar. 2,2′,7,7′-tetrakis(*N*,*N*-di-p-methoxyphenylamino)−9,9′-spirobifluorene (spiro-OMeTAD; ≥99.5%) was purchased from Lumtec. Cesium carbonate (Cs_2_CO_3_; 99.9%), oleylamine (OAm; technical grade, 70%), oleic acid (OA; technical grade, 90%), 1-octadecene (ODE; technical grade, 90%), octane (anhydrous, ≥99%), hexane (reagent grade, ≥95%), methyl acetate (MeOAc; anhydrous, 99.5%), lead nitrate (Pb(NO_3_)_2_; 99.999%), ethyl acetate (EtOAc; anhydrous, 99.8%), formamidinium acetate (FA-acetate, 99%), titanium ethoxide (≥97%), hydrochloric acid (HCl; 37% in water), chlorobenzene (anhydrous, 99.8%), 4-tert-butylpyridine (4-TBP; 96%), toluene (anhydrous, 99.8%), dimethylformamide (DMF), dimethyl sulfoxide (DMSO), and acetonitrile (anhydrous, 99.8%) were purchased from Sigma-Aldrich and used as received unless otherwise specified.

### CsPbI_3_ QDs synthesis and purification

The synthesis was performed following the method reported in our previous publications with slight modification^11,25^. First, 0.407 g of Cs_2_CO_3_ was added in 20 mL of ODE containing 1.25 mL of OA and degassed at 150 °C for 20 min under vacuum in a 100-mL three-neck flask. After Cs_2_CO_3_ was absolutely dissolved in the solution, the Cs-oleate precursor was completed and kept in N_2_ at 150 °C until needed. Subsequently, 0.5 g of PbI_2_ and 25 mL of ODE were mixed in a 100-mL three-neck flask at 120 °C for 10 min under vacuum. A preheated mixture of OA and OAm (130 °C, 2.5 mL each) was injected into the PbI_2_ solution and the reaction flask was kept at 120 °C until a clear solution was formed. Then the reaction flask was heated to 185 °C under flowing N_2_. Once 2 mL of the Cs-oleate precursor was immediately injected into the reaction flask, the mixture was quenched in an ice bath. After cooling to room temperature, the colloidal solution was mixed with 70 to 80 mL of MeOAc and then centrifuged at 7500 rpm for 5 min. The precipitated QDs were dispersed in 5 mL of hexane and re-precipitated by adding about 5 mL of MeOAc. After centrifugation (7500 rpm, 5 min), the resulting precipitate was redispersed in 15 mL of hexane and stored in refrigerator at 4 °C. To remove excess Cs-oleate and Pb-oleate, the QD solution was centrifuged at 7500 rpm for 5 min after 24 hours of storage in refrigerator. The supernatant was filtered through a 0.45-μm nylon filter and ready for used.

### FAPbI_3_ QDs synthesis and purification

FAPbI_3_ QDs was synthesized and purified following the previously reported method^26^. The FA-oleate precursor was prepared by first degassing a mixture of 0.521 g of FA-acetate and 10 mL of OA in a 100-mL three-neck flask under a vacuum at 120 °C for 20 min. Then N_2_ was introduced in the reaction flask and the clear reaction solution was yielded. After cooling to 90 °C, the FA-oleate precursor was kept in N_2_ and ready for injection. To prepare PbI_2_ solution, 0.344 g of PbI_2_ was added into 20 mL of ODE and degassed at 120 °C for 20 min under vacuum in a 100-mL three-neck flask. A preheated mixture of OA and OAm (130 °C, 3 mL each) was injected into the PbI_2_ solution and the reaction flask was kept at 120 °C until a clear solution was formed. Purging the flask with N_2_, the PbI_2_ solution was cooled down and kept at 80 °C. FA-oleate precursor (5 mL) was immediately injected into the PbI_2_ solution. The mixture was quenched in an ice bath after 15 s. MeOAc (9 mL) was added into the colloidal solution after cooling to room temperature and then centrifuged at 8000 rpm for 30 min. The precipitated QDs was dispersed in 9 mL of toluene. Around 10 mL of MeOAc was added into the QDs solution and then it was centrifuged at 8000 rpm for 10 min. The resulting precipitate was redispersed in 10 mL of octane and stored in refrigerator at 4 °C for at least 24 h. Before use, the QDs solution was centrifuged at 7500 rpm for 5 min and the precipitate was decarded. The supernatant was filtered through a 0.45-μm nylon filter.

### Preparation of Cs_1−*x*_FA_*x*_PbI_3_ alloys

Colloidal solutions of CsPbI_3_ and FAPbI_3_ QDs were dispersed in octane, respectively. To obtain similar concentrations of these two solutions, the absorption spectra were measured, and each solution had a similar optical density near the band edge. After the concentrations were calibrated, these two colloidal solutions were mixed in different volume ratios to yield Cs_1-*x*_FA_*x*_PbI_3_ QDs with the desired Cs/FA stoichiometry and then stored at room temperature for at least 48 h^26^.

### Fabrication of QD films

Coupled Cs_1-*x*_FA_*x*_PbI_3_ QD films (where *x* = 0, 0.25, 0.50, 0.75, or 1.0) were deposited following previously reported methods^26^ with slight modifications. Deposition of all QD films was carried out in a humidity-controlled box (relative humidity, 20–25%). First, saturated Pb(NO_3_)_2_ in MeOAc solution and FAI in EtOAc solution were prepared. Each layer of Cs_1-*x*_FA_*x*_PbI_3_ QD was deposited by spin-coating at 1000 rpm for 20 s and 2000 rpm for 5 s. Subsequently, the film was swiftly soaked into the Pb(NO_3_)_2_ solution and then rinsed in the neat MeOAc solution. A total film with ~300 nm of thickness was obtained by repeating the process of spin-coating QDs and soaking in the solution four times. To achieve a heterojunction structure, Cs_1-*x*_FA_*x*_PbI_3_ QDs with different Cs/FA stoichiometry was deposited by following different sequences. Finally, the film was dipped in FAI solution for 10 s and rinsed in the neat MeOAc solution^10,12^.

### Fabrication of PV devices

All of PV devices was fabricated by following previous literatures with slight modification^10,11^. Patterned indium-doped tin oxide (ITO) glass was cleaned by sonicating in isopropanol for 30 min and treated with UV-ozone for 5 min. An ~50 nm compact TiO_2_ layer was deposited by spin-coating a clear solution of TiO_2_ at 3000 rpm for 20 s ;and then annealing at 450 °C for 30 min. The TiO_2_ solution was synthesized by a sol-gel method from a precursor prepared by mixing 5 mL of EtOH, two drops of HCl, 125 mL of deionized water, and 375 mL of titanium ethoxide. The QD absorber layer was deposited following the procedure described above. Spiro-OMeTAD films were deposited by spin-coating at 5000 rpm for 30 s from a solution containing 72.3 mg spiro-OMeTAD, 28.8 μL 4-TBP and 17.5 μL of a bis(trifluoromethane)sulfonimide lithium salt (LiTFSI) stock solution (520 mg mL^−1^ in acetonitrile) dissolved in 1 mL of chlorobenzene. All the spin-coating processes were carried out under ambient conditions unless otherwise specified. MoO_*x*_ was evaporated at a rate of 0.1 to 0.5 Å s^−1^ at a base pressure lower than 2 × 10^−7^ torr, resulting in a total thickness of 15 nm. Al electrodes were deposited at a rate of 0.5 to 2.0 Å s^−1^ for a total thickness of 200 nm. The encapsulation of devices was processed in a N_2_-filled glovebox by sealing the active area to another piece of glass with polyisobutylene tape.

### Characterization of QD solutions and films

TEM images were obtained using a FEI Tecnai F20 electron microscope with 200 kV accelerating voltage. To determine elemental and isotopic distributions in solids, as well as the structure and composition of organic materials, time-of-flight secondary ion mass spectrometry (ToF-SIMS) is a powerful analytical technique to investigate HPSC materials and devices^13,27^. An ION-ToF ToF-SIMS V spectrometer was utilized to depth-profile the Cs_1-*x*_FA_*x*_PbI_3_ QD films. Analysis was carried out utilizing a 3-lens 30 kV BiMn primary ion gun, and the Bi_3_^+^ primary-ion beam (operated in bunched mode; 21 ns pulse width, analysis current 0.7 pA) was scanned over a 25 × 25 micron area. Depth Profiling was completed with a 1 kV oxygen ion sputter beam (7 nA sputter current) rastered over a 150 × 150 micron area. All spectra during profiling were recorded at or below a primary ion dose density of 1 × 10^12^ ions cm^−2^ to remain at the static-sims limit.

Transient absorption spectra (TA) were recorded using a Continuum Integra-C laser, with an output of 800 nm at 1 kHz. The 800 nm beam was directed into a Palitra optical parametric amplifier to generate pump pulse (about 150 fs) at 400 nm and was modulated at 500 Hz through an optical chopper to block every other laser pulse. Femtosecond TA spectra were collected using Helios spectrometer (Ultrafast Systems). A small amount of 800 nm light was used to pump a sapphire crystal to create 450–800 nm probe light for TA.

For time-resolved microwave conductivity (TRMC) measurements, the perovskite QD films deposited onto pre-cleaned quartz substrates (1 cm × 2.5 cm × 1 mm) were photoexcited through the quartz side of the substrate with 650 nm (5 ns pulse width) pules at 10 Hz from an optical parametric oscillator (Continuum Panther) pumped by the 355 nm harmonic of a Q-switched Nd:YAG laser (Continuum Powerlite). The transient change in photoconductance, Δ*G(t)*, was recorded via transient change in the microwave power, Δ*P(t)*, due to absorption of microwaves (ca. 9 GHz) by mobile photogenerated charge carriers in the sample. Δ*G(t)* was calculated by:

1 $${\mathrm{\Delta }}G\left( t \right) = \left( { - \frac{1}{K}} \right)\left( {\frac{{{\mathrm{\Delta }}P\left( t \right)}}{P}} \right)$$

where *K* is a calibration factor experimentally determined from the dielectric properties of the QD films and the resonance characteristics of the microwave cavity. The end-of-pulse (peak) photoconductance, Δ*G*_peak_, is related to the yield mobility product (*φ*Σ*μ*) using the equation:

2 $${\mathrm{\Delta }}G_{{\mathrm{peak}}} = \beta q_{\mathrm{e}}N\left( {\mu _{\mathrm{e}} + \mu _{\mathrm{h}}} \right) = \beta q_{\mathrm{e}}I_0F_{\mathrm{A}}\varphi \Sigma \mu$$

where *β* = 2.2 and is the ratio of the interior dimensions of the waveguide, *φ* is the yield of free-carrier generation, *q*_e_ is the electronic charge, *N* is the number of photogenerated charge-carrier pairs, *μ*_e_ and *μ*_h_ are the electron and hole mobilities (termed *Σμ*), *I*_0_ is the incident photon flux of the excitation laser pulse, and *F*_A_ is the fraction of photons (650 nm) absorbed by the QD films. Pump excitation intensities were decreased until peak photoconductivity values and transient lifetime were intensity independent, provided sufficient signal-to-noise. At 650 nm, generation of charges can be uniform throughout the vertical cross section of the films, thus further mitigating higher-order recombination processes. Hence, we extracted the carrier mobilities of the QD films from this linear response region of the data.

XPS and UPS measurements were performed on a Kratos Axis Nova instrument, using monochromated Al-Kα (hv = 1486.7 eV), and He-I (hv = 21.22 eV) excitation sources, respectively. The spectrometer binding-energy (BE) scale was calibrated by measuring valence-band and core-level spectra from sputter-cleaned Au and Cu foils (*E*_F_ = 0.00 eV, Au 4*f*_7/2_ = 83.96 eV, and Cu 2*p*_3/2_ = 932.62 eV)^28^. XPS/UPS spectra were acquired at pass energy *E*_pass_ = 20 eV. The reference energy levels for spiro-OMeTAD and TiO_2_ were taken from literature^29^.

### Characterization of PV devices

Devices were tested on a Newport Oriel Sol3A solar simulator with xenon lamp in the glove box at room temperature. The intensity of the solar simulator was calibrated to 100 mW cm^−2^ AM1.5 G with a KG5 filtered Si reference solar cell that was certified by NREL PV Performance Characterization Team and the spectral mismatch factor was minimized to 0.9923. *J*–*V* scans were measured from forward bias to reverse bias step and from reverse bias to forward (bias size: 100 mV, delay time: 10 ms, number of power line cycles: 0.1, respectively. The devices were masked with a black metal aperture to define an active area of 0.059 cm^2^. The stabilized power output (SPO) of devices was measured by holding the illuminated devices at a constant voltage near the maximum power point of the *J*–*V* scan and recording the continuous current output in the meantime. External quantum efficiency (EQE) measurements were performed utilizing a Newport Oriel IQE200.

To obtain the cross-sectional TEM images of devices, a Pt capping layer was deposited on the surface of devices to protect the thin-film structure. TEM foils were prepared by the standard focused ion beam (FIB) lift-out technique and then the final thickness was reduced to less than <100 nm by a Ga ion milling. To remove preparation damage on the TEM foils, a Fischione Nanomill was used and a low energy cleaning was carried out at 500 eV under vacuum of 10^−7^ torr at −170 °C for ±10°. TEM observations were operated on a FEI Tecnai F20 TEM at an acceleration voltage of 200 kV. Chemical analysis was performed with energy-dispersive X-ray spectrometry (EDS) in the high-angle annular dark-field (HAADF)-scanning TEM (STEM) mode.

## Supplementary information

Supplementary Information

Reporting Summary

## Data Availability

Data that support the plots within this work are available from the corresponding author upon reasonable request.
