# Supplementary material for: High efficiency perovskite quantum dot solar cells with charge separating heterostructure
Source: Nat Commun. 2019 Jun 28;10:2842. doi: 10.1038/s41467-019-10856-z (PMC6599010; doi:10.1038/s41467-019-10856-z)
Supplement: Supplementary file 1 — Supplementary Information [file 41467_2019_10856_MOESM1_ESM.pdf]

High Efficiency Perovskite Quantum Dot Solar Cells with Charge Separating Heterostructure

Authors: Qian Zhao,[1,2,3] Abhijit Hazarika,[2] Xihan Chen,[2] Steve P. Harvey,[2] Bryon W. Larson,[2] Glenn R. Teeter,[2] Jun Liu,[2] Tao Song,[2] Chuanxiao Xiao,[2] Liam Shaw,[4] Minghui Zhang,[1] Guoran Li,[3] Matthew C. Beard,[2] Joseph M. Luther[2]*

Affiliations:

[1]College of Chemistry, Nankai University, Tianjin 300071, China

[2]National Renewable Energy Laboratory, Golden, CO 80401, USA

[3]Institute of New Energy Chemistry Material, Nankai University, Tianjin 300350, China

[4]Warren Wilson College, Asheville, NC 28815, USA

*Correspondence to: joey.luther@nrel.gov

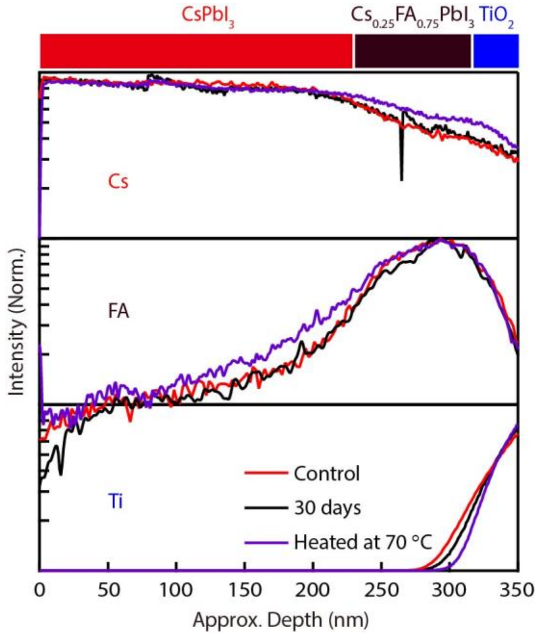

Supplementary Figure 1 | The stability of heterojunction structure. ToF-SIMS depth profile is used to detail the stability of the interface location for films of $CsPbI_3/Cs_{0.25}FA_{0.75}PbI_3/TiO_2$. The intended structure is indicated in the bar above the plots. The FA, Cs and Ti signals from perovskite QD layer and TiO_2 are shown. Compared to the fresh control sample, an aged sample was stored in a N_2 glovebox for 30 days and a heated sample was put on a hotplate at 70 ºC for 6 hours. There is no noticeable change between the control and the 30-days sample in depth profile. However, the slopes of Cs and FA signals are slightly reduced at the boundary between the $CsPbI_3$ and $Cs_{0.25}FA_{0.75}PbI_3$ layers in the heated sample compared with the control.

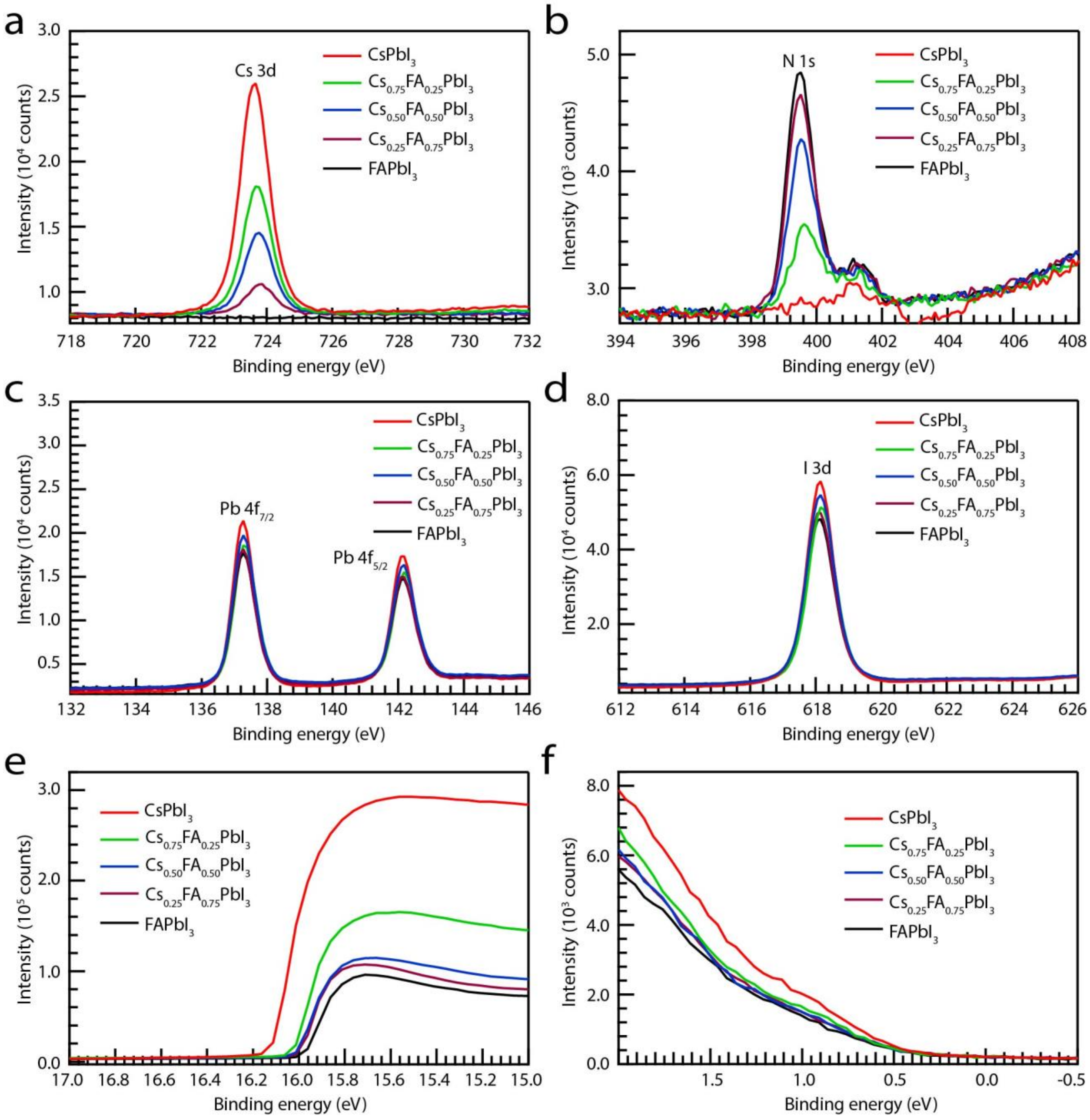

Supplementary Figure 2 | XPS and UPS spectra of pure $CsPbI_3$, $Cs_{0.75}FA_{0.25}PbI_3$, $Cs_{0.5}FA_{0.5}PbI_3$, $Cs_{0.25}FA_{0.75}PbI_3$, and $FAPbI_3$ QD films as the same as the films used in devices. **a-d**, The Cs 3d, N 1s, Pb 4f and I 3d photoemission spectra of these pure QD films, respectively, which provides experimental evidences of the compositions of $CsPbI_3$, $Cs_{0.75}FA_{0.25}PbI_3$, $Cs_{0.5}FA_{0.5}PbI_3$, $Cs_{0.25}FA_{0.75}PbI_3$, and $FAPbI_3$ QD films. **e-f**, UPS spectra of these pure QD films showing the enlarged views of the secondary electron tail threshold and top valence band regions, respectively.

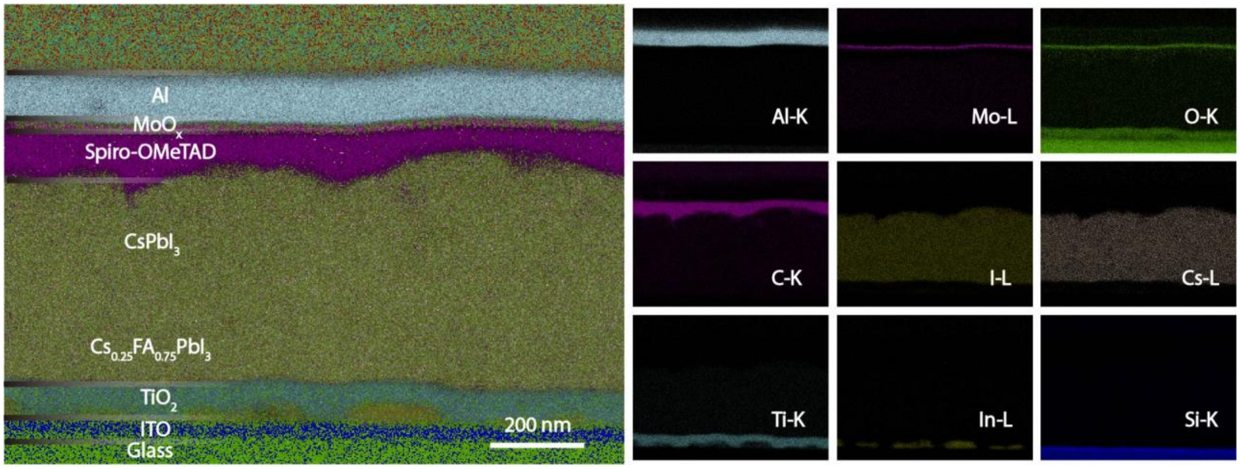

Supplementary Figure 3 | Chemical analysis in cross-sectional STEM-HAADF images of devices. Al-K, Mo-L, O-K, C-K, I-L, Cs-L, Ti-K, In-L and Si-K edge EDS elemental mapping of the cross-section of devices, confirming the position of each film within devices.

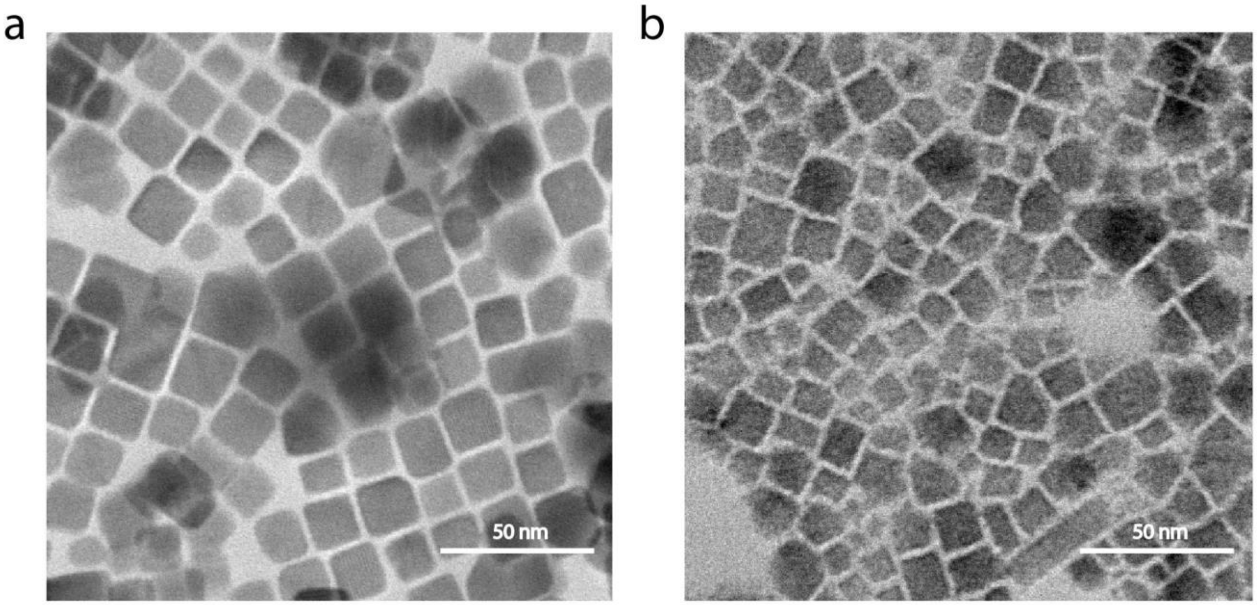

Supplementary Figure 4 | Morphology of QDs. a, TEM image of $CsPbI_3$ QDs. **b,** TEM image of $Cs_{0.25}FA_{0.75}PbI_3$ QDs. Scale bar, 50 nm.

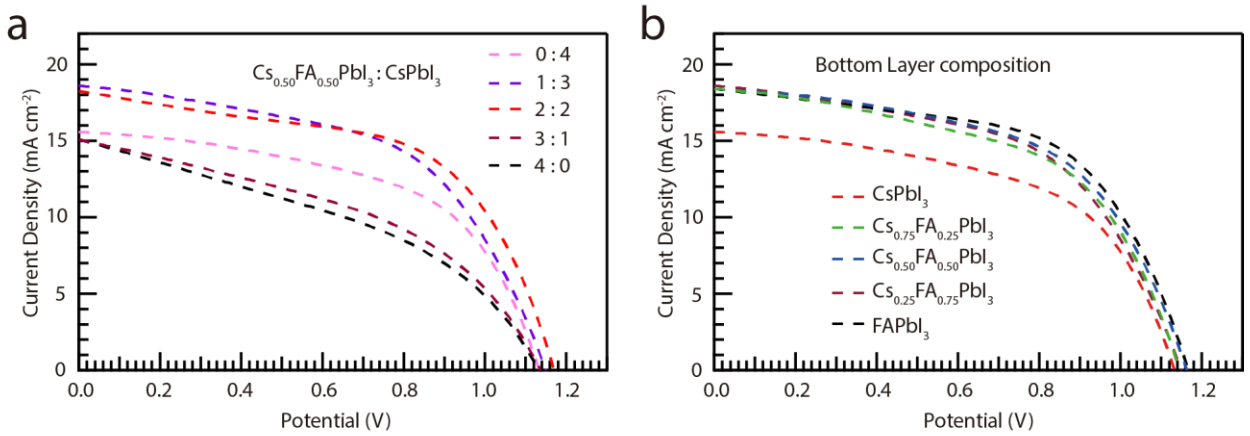

Supplementary Figure 5 | *J-V* curves measured by forward scans (from short circuit to open circuit). a, *J-V* curves from forward scans for devices with different thickness ratio of $Cs_{0.25}FA_{0.75}PbI_3$ layer to $CsPbI_3$ layer in the perovskite QD absorber. **b**, *J-V* curves from forward scans for devices with different compositions of the bottom layer in the perovskite QD absorber where the thickness ratio of the mixed-cation $Cs_xFA_{1-x}PbI_3$ QD layers to $CsPbI_3$ QD layers is 1:3 in all cases.

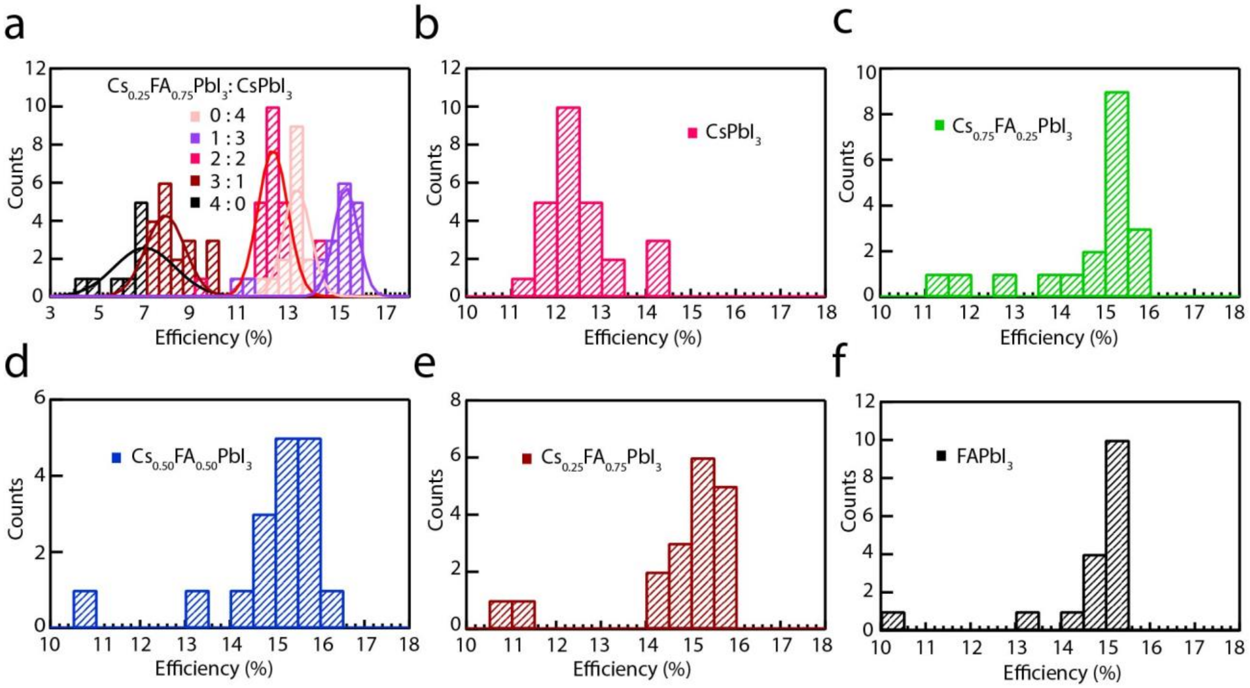

Supplementary Figure 6 | Histograms of conversion efficiency from SPO. a, Histograms of PCE from SPO for devices with different thickness ratio of $Cs_{0.25}FA_{0.75}PbI_3$ layer to $CsPbI_3$ layer in the perovskite QD absorber. Data are from 19, 20, 27, 19 and 18 devices for 0:4, 1:3, 2:2, 3:1 and 4:0 of thickness ratio of $Cs_{0.25}FA_{0.75}PbI_3$ layer to $CsPbI_3$ layer, respectively. **b-f**, Histograms of PCE from SPO for devices with $CsPbI_3$, $Cs_{0.75}FA_{0.25}PbI_3$, $Cs_{0.5}FA_{0.5}PbI_3$, $Cs_{0.25}FA_{0.75}PbI_3$, and $FAPbI_3$ of the bottom layer in the perovskite QD absorber where the thickness ratio of the mixed-cation $Cs_xFA_{1-x}PbI_3$ QD layers to $CsPbI_3$ QD layers is 1:3 in all cases, respectively. Data are from 27, 19, 17, 18 and 17 devices for $CsPbI_3$, $Cs_{0.75}FA_{0.25}PbI_3$, $Cs_{0.5}FA_{0.5}PbI_3$, $Cs_{0.25}FA_{0.75}PbI_3$, and $FAPbI_3$ of the bottom layer in the perovskite QD absorber, respectively.

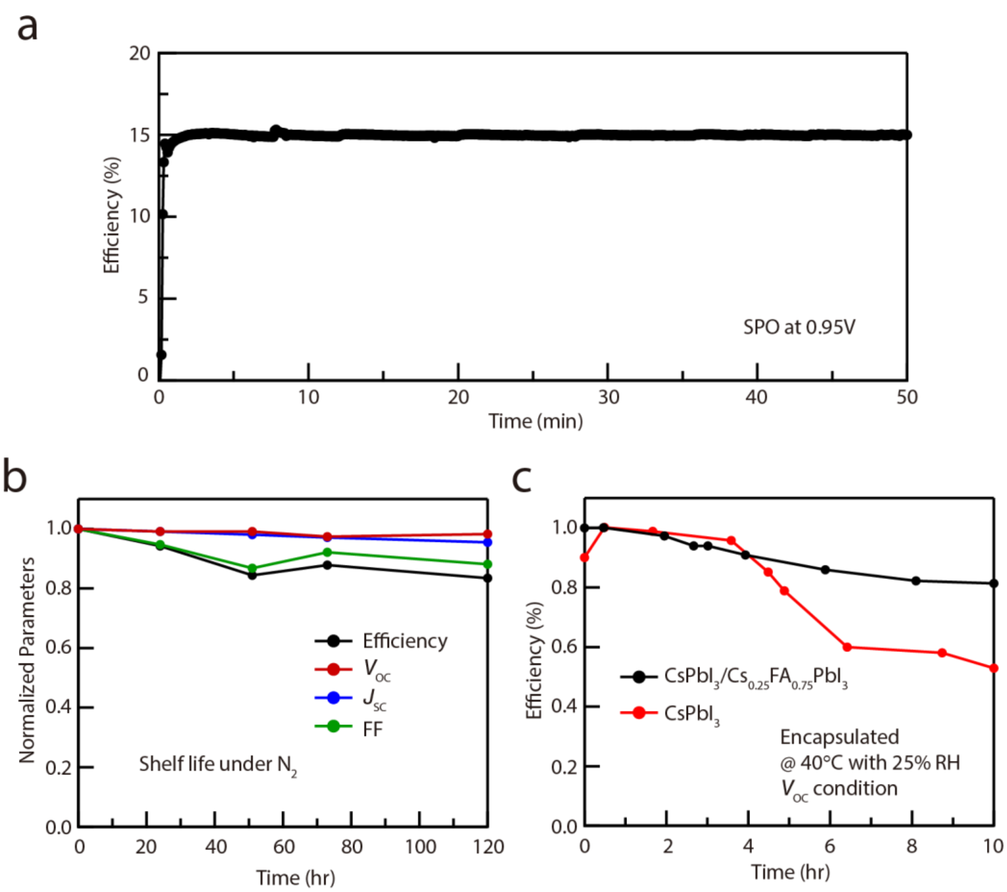

Supplementary Figure 7 | Stability measurement of solar cells. a, SPO of devices with the heterojunction (1:3 thickness ratio of $Cs_{0.25}FA_{0.75}PbI_3$ layer to $CsPbI_3$ layer in the perovskite QD absorber) was measured by recording the continuous current output per 5 s at 0.95 V over 50 min of AM1.5 100 mW cm^{-2} solar illumination. **b**, Evolution of normalized photovoltaic parameters for devices with the heterojunction (1:3 thickness ratio of $Cs_{0.25}FA_{0.75}PbI_3$ layer to $CsPbI_3$ layer in the perovskite QD absorber). The aged devices were stored in the dark for 120 hours under a N_2 atmosphere. **c**, Evolution of normalized efficiency for devices made with the heterojunction (1:3 thickness ratio of $Cs_{0.25}FA_{0.75}PbI_3$ layer to $CsPbI_3$ layer in the perovskite QD absorber) and pure $CsPbI_3$. The devices were encapsulated and measured at 40 ºC and 25 % of humidity over 10 hours of constant illumination.

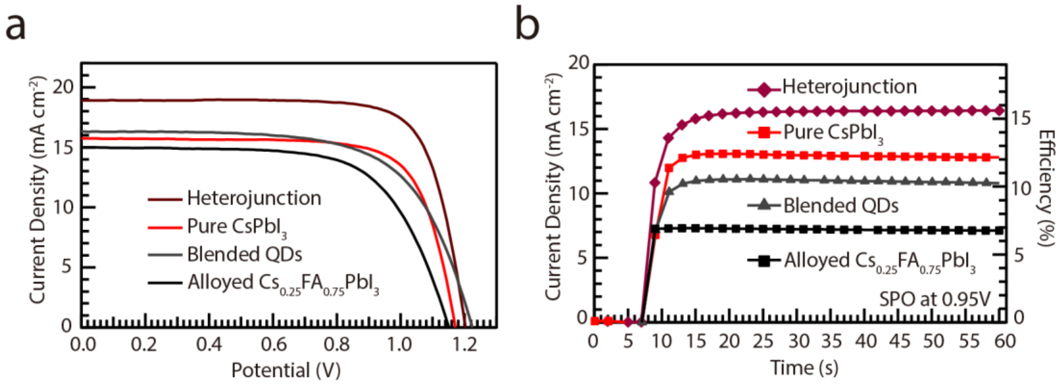

Supplementary Figure 8 | Photovoltaic performance of solar cells with the blended-QD films as a reference. a,b, *J-V* curves recorded by reverse scans and SPO at 0.95 V of devices made with the heterojunction (1:3 thickness ratio of $Cs_{0.25}FA_{0.75}PbI_3$ layer to $CsPbI_3$ layer in the perovskite QD absorber), pure $CsPbI_3$, blended QDs (the QD solution was prepared by blending $Cs_{0.25}FA_{0.75}PbI_3$ with $CsPbI_3$ QDs with the ratio of 1:3) and alloyed $Cs_{0.25}FA_{0.75}PbI_3$. The devices fabricated with the blended QDs have a V_{oc} of 1.22 V, J_{sc} of 16.30 mA/cm^{-2}, FF of 0.65, PCE of 13.01% (from reverse scan) and SPO of 10.16% at 0.95V.

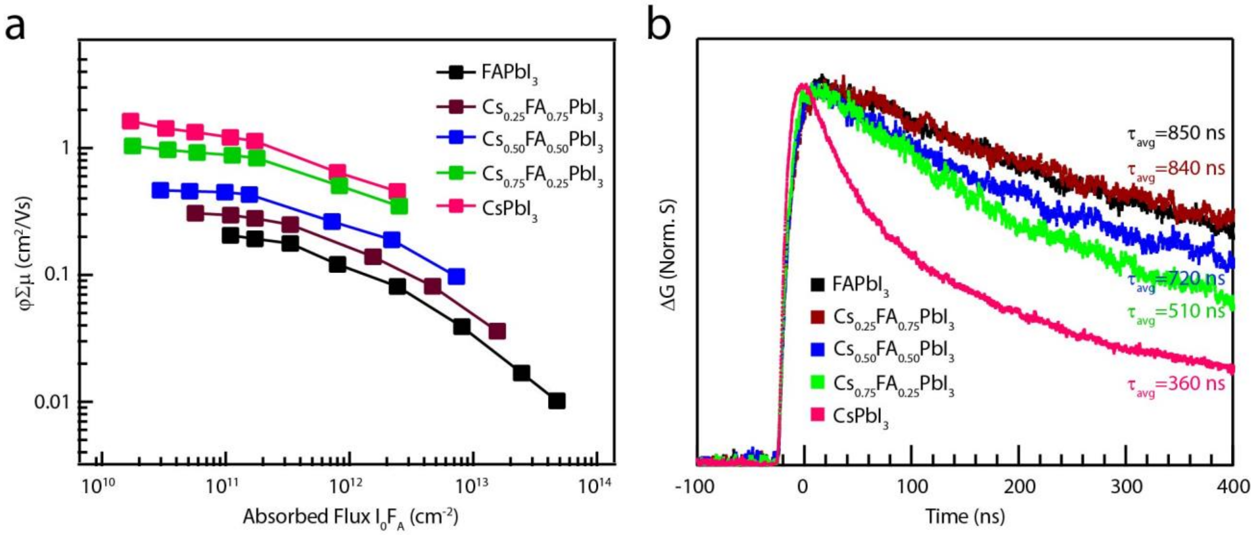

Supplementary Figure 9 | TRMC measurement on pure $CsPbI_3$, $Cs_{0.75}FA_{0.25}PbI_3$, $Cs_{0.5}FA_{0.5}PbI_3$, $Cs_{0.25}FA_{0.75}PbI_3$, and $FAPbI_3$ QD films. a, $\varphi\sum\mu$ transients shown over ca. four orders of magnitude excitation intensity at 620 nm. Mobility values for each of the QD thin films are extracted by assuming an internal quantum conversion efficiency of 1 at absorbed flux values at or below 10^{11} cm^{-2}. The excitation wavelength is chosen close to the band edge, ensuring a more uniform charge generation profile throughout the cross section of the film. The mobility of QD films is decreased by increasing the ratio of FA alloyed in these QDs. **b**, Normalized photoconductivity transients for each of the alloy compositions over 400 ns. Average weighted lifetime values are extracted from bi-exponential fits of the photoconductivity transients according to $(A1Tau1+A2Tau2)/(A1+A2)$. Clearly, alloying FA in these dots improves the carrier lifetime. The lifetimes of free carriers for $CsPbI_3$, $Cs_{0.75}FA_{0.25}PbI_3$, $Cs_{0.5}FA_{0.5}PbI_3$, $Cs_{0.25}FA_{0.75}PbI_3$, and $FAPbI_3$ QDs are 360 ns, 510 ns, 720 ns, 840 ns and 850 ns, respectively.

Supplementary Table 1 | Photovoltaic parameters of solar cells. *J*-*V* parameters from forward scans for devices with different thickness ratio of $Cs_{0.25}FA_{0.75}PbI_3$ layer to $CsPbI_3$ layer in the perovskite QD absorber.

Thickness ratio ($Cs_{0.25}FA_{0.75}PbI_3$: $CsPbI_3$)	V_{oc} (V)	J_{sc} (mA cm^{-2})	FF (%)	PCE (%)
0 : 4	1.13	15.58	55	9.63
1 : 3	1.15	18.61	54	11.46
2 : 2	1.17	18.26	56	12.05
3 : 1	1.14	15.07	43	7.39
4 : 0	1.13	15.09	40	6.81

Supplementary Table 2 | Photovoltaic parameters of solar cells. *J-V* parameters and conversion efficiency of devices with different compositions of the bottom layer in the perovskite QD absorber where the thickness ratio of the mixed-cation $Cs_xFA_{1-x}PbI_3$ QD layers to $CsPbI_3$ QD layers is 1:3 in all cases.

Bottom layer composition	From forward scans				From reverse scans				SPO (%)
	V_{oc} (V)	J_{sc} (mA cm^{-2})	FF (%)	PCE (%)	V_{oc} (V)	J_{sc} (mA cm^{-2})	FF (%)	PCE (%)	
$CsPbI_3$	1.13	15.58	55	9.63	1.17	15.75	74	13.67	12.15
$Cs_{0.75}FA_{0.25}PbI_3$	1.15	18.37	54	11.33	1.21	18.57	74	16.67	15.74
$Cs_{0.50}FA_{0.50}PbI_3$	1.16	18.37	55	11.79	1.21	18.53	75	16.73	15.62
$Cs_{0.25}FA_{0.75}PbI_3$	1.15	18.61	54	11.46	1.20	18.91	76	17.39	15.57
$FAPbI_3$	1.17	18.38	57	12.32	1.21	18.84	76	17.32	15.21
