# Supplementary material for: High efficiency perovskite quantum dot solar cells with charge separating heterostructure
Source: Nat Commun. 2019 Jun 28;10:2842. doi: 10.1038/s41467-019-10856-z (PMC6599010; doi:10.1038/s41467-019-10856-z)
Supplement: Supplementary file 2 — Reporting Summary [file 41467_2019_10856_MOESM2_ESM.pdf]

nature research

Corresponding author(s): *Double-blind peer review submissions: write DBPR and your manuscript number here instead of author names.*

Solar Cells Reporting Summary

Nature Research wishes to improve the reproducibility of the work that we publish. This form is intended for publication with all accepted papers reporting the characterization of photovoltaic devices and provides structure for consistency and transparency in reporting. Some list items might not apply to an individual manuscript, but all fields must be completed for clarity.

For further information on Nature Research policies, including our data availability policy, see Authors & Referees.

Experimental design

Please check: are the following details reported in the manuscript?

1. Dimensions

Item	Yes/No	Details
Area of the tested solar cells	☒ Yes ☐ No	Clearly stated in Characterization of PV devices in Methods
Method used to determine the device area	☒ Yes ☐ No	Clearly stated in Characterization of PV devices in Methods

2. Current-voltage characterization

Item	Yes/No	Details
Current density-voltage (J-V) plots in both forward and backward direction	☒ Yes ☐ No	Clearly stated in Characterization of PV devices in Methods
Voltage scan conditions<br>*For instance: scan direction, speed, dwell times*	☒ Yes ☐ No	Clearly stated in Characterization of PV devices in Methods
Test environment<br>*For instance: characterization temperature, in air or in glove box*	☒ Yes ☐ No	All cells measured in nitrogen glovebox and it's clearly stated in Characterization of PV devices in Methods
Protocol for preconditioning of the device before its characterization	☐ Yes ☒ No	The devices were tested without any preconditions.
Stability of the J-V characteristic<br>*Verified with time evolution of the maximum power point or with the photocurrent at maximum power point; see ref. 7 for details.*	☒ Yes ☐ No	Stated in manuscript and Stabilized Power Output plotted in figure 3

3. Hysteresis or any other unusual behaviour

Item	Yes/No	Details
Description of the unusual behaviour observed during the characterization	☒ Yes ☐ No	It can be found in description of Fig.3. (Paragraph 7)
Related experimental data	☒ Yes ☐ No	See Fig.3 and Extended data Fig. 4. Parameters were presented in Tables

4. Efficiency

Item	Yes/No	Details
External quantum efficiency (EQE) or incident photons to current efficiency (IPCE)	☒ Yes ☐ No	EQE provided in Figure 2.
A comparison between the integrated response under the standard reference spectrum and the response measure under the simulator	☒ Yes ☐ No	While not expressed directly in the manuscript, the integrated EQE is 97% of the measured Jsc.
For tandem solar cells, the bias illumination and bias voltage used for each subcell	☐ Yes ☒ No	No tandem cells presented in this work

5. Calibration

Item	Yes/No	Details
Light source and reference cell or sensor used for the characterization	☒ Yes ☐ No	It can be found in Characterization of PV devices in Methods
Confirmation that the reference cell was calibrated and certified	☒ Yes ☐ No	It can be found in Characterization of PV devices in Methods

November 2017

Calculation of spectral mismatch between the reference cell and the devices under test

- [x] Yes
- [ ] No

The spectral mismatch was calculated (0.9923) and It can be found in Characterization of PV devices in Methods.

6. Mask/aperture

Size of the mask/aperture used during testing

- [x] Yes
- [ ] No

All devices have active area of 0.11 cm2 but are masked with an aperture to 0.059 cm2. Clearly stated in Characterization of PV devices in Methods

Variation of the measured short-circuit current density with the mask/aperture area

- [ ] Yes
- [x] No

An aperture/mask was always applied during measurement

7. Performance certification

Identity of the independent certification laboratory that confirmed the photovoltaic performance

- [ ] Yes
- [x] No

No solar cell efficiency world record is claimed in this work. Our group's cells have been certified in past to verify our measurements are in line with accredited laboratories

A copy of any certificate(s)
Provide in Supplementary Information

- [ ] Yes
- [x] No

No solar cell efficiency world record is claimed in this work.

8. Statistics

Number of solar cells tested

- [x] Yes
- [ ] No

See Extended Data Fig.5

Statistical analysis of the device performance

- [x] Yes
- [ ] No

See Extended Data Fig.5

9. Long-term stability analysis

Type of analysis, bias conditions and environmental conditions
For instance: illumination type, temperature, atmosphere humidity, encapsulation method, preconditioning temperature

- [x] Yes
- [ ] No

Long term stability has been performed in various ways and is stated clearly in the Extended Data
